# DNA extraction from recently fertilised Atlantic salmon embryos for use in microsatellite validation of triploidy

**DOI:** 10.1371/journal.pone.0292319

**Published:** 2023-10-04

**Authors:** Callum Howard, John B. Taggart, Caroline R. Bradley, Alejandro P. Gutierrez, John F. Taylor, Paulo A. Prodöhl, Herve Migaud, Michaël Bekaert

**Affiliations:** 1 Institute of Aquaculture, University of Stirling, Stirling, United Kingdom; 2 School of Biological Sciences, Queen’s University Belfast, Belfast, United Kingdom; University of Helsinki: Helsingin Yliopisto, FINLAND

## Abstract

The current methods used for producing triploid Atlantic salmon are generally reliable but not infallible, and each batch of triploids must be validated to ensure consumer trust and licensing compliance. Microsatellites have recently been shown to offer a cheaper and more convenient alternative to traditional flow cytometry for triploidy validation in a commercial setting. However, incubating eggs to at least the eyed stage for microsatellite validation poses challenges, such as reduced quality and performance of triploids produced from later eggs in the stripping season. To address these issues, we propose another option: extracting DNA from recently fertilised eggs for use in conjunction with microsatellite validation. To achieve this, we have developed an optimized protocol for HotSHOT extraction that can rapidly and cheaply extract DNA from Atlantic salmon eggs, which can then be used for triploidy validation through microsatellites. Our approach offers a simpler and more cost-effective way to validate triploidy, without the need for skilled dissection or expensive kits.

## Introduction

The current methods used for producing triploid Atlantic salmon (*Salmo salar* L. 1758) are generally reliable, but not infallible [1]. Each batch of triploids must be validated to ensure consumer trust and licensing compliance. While traditionally flow cytometry or blood smears have been used for validation, recent studies have shown that microsatellites can also be reliably used for this purpose in a commercial setting [2], and at a lower cost than flow cytometry [3].

Flow cytometry and blood smears require eggs to be incubated until the eyed stage, at which point sufficient numbers of erythrocytes are present to allow verification to take place. Previous studies involving microsatellite validation have also incubated eggs to at least the eyed stage [2, 4, 5]. Incubation to this stage poses issues however, producers are faced with two options; produce triploids as normal, verifying once eyeing is reached but running the risk that, should the triploidy procedure have failed, the stripping season may be over and batches cannot be re-made. Even if the stripping season is not over, there is good evidence that triploids produced from eggs later in the stripping season are of worse quality and perform worse [6]. The other option is to accelerate a sub-batch of eggs through incubation at higher temperatures, this of course requires more resources, although increases the likelihood that batches can be re-made within the stripping season. To address these challenges, we propose a third option: extracting DNA from recently fertilised eggs for use in microsatellite validation. This approach would allow orders to be remade without the need for sub-batch incubation, and the DNA extraction method should be cheap and reliable.

Extracting usable DNA from Atlantic salmon eggs can be challenging due to the low amount of embryonic DNA and the lipid-rich nature of the egg [7, 8]. Dissecting the embryo from the rest of the egg or using expensive kits such as the Qiagen DNEasy kit are common approaches [9, 10], but they are time-consuming and costly. More traditional approaches such as salting out or phenol-chloroform are laborious, and cruder methods such as HotSHOT (Hot sodium hydroxide and TRIS) has been shown to extract DNA rapidly from fish embryos [11–13], but have not yet been shown to be effective on salmonid eggs.

We developed an optimized HotSHOT protocol for DNA extraction from Atlantic salmon eggs, providing a simple, cost-effective, and efficient method for validating triploidy using microsatellites. This method can be easily integrated into commercial salmon production, allowing for more reliable validation of triploids and greater consumer trust.

## Materials and methods

The detailed protocol described in this article is published in protocols.io, https://dx.doi.org/10.17504/protocols.io.kqdg3x93pg25/v1, and is included for printing with this article as S1 Protocol.

### Ethics statement

Animal handling and collection in this study was carried out in accordance with the UK Animals (Scientific Procedures) Act 1986 Amended Regulations (SI 2012/3039) and the work was approved by the University of Stirling Ethics Committee (Animal Welfare and Ethics Review Board).

### Tissue collection

This protocol was established using triploid Atlantic salmon eggs stored in ethanol, representing developmental stages at 26, 44, 61, and 78 degree-days post-fertilisation (corresponding to approximately 5, 9, 12, and 16% of development to hatching). The eggs were submerged in a 10:1 ethanol-to-egg volume ratio, with 100% ethanol. These preserved eggs were stored at a temperature range of 4–8°C for a duration ranging from 3 days to 1 year.

### DNA extraction

- If eggs stored in ethanol, remove using forceps and place on clean tissue to remove excess ethanol.
- Place the batch of eggs in a beaker of Tris-HCl (5 mM, pH 8) for 15 minutes
- Remove the eggs from the beaker and remove excess liquid with clean tissue
- For low throughput needs the eggs can then be placed into individual 1.5ml screw cap tubes, for high throughput needs the eggs can be places, one per well, into a 2 mL deep 96-well plate.
- Pierce the chorion by applying pressure using the end of the forceps (Between eggs, the forceps must be wiped clean before being sterilised using 100% ethanol and ddH_2_O).
- Add 400 μL alkaline lysis buffer to each tube/well and seal.
- Invert 5 times, and placed into either a heat block or a laboratory oven running at 90°C for 30 minutes.
- Remove and place on ice for 5 minutes.
- Unseal and add an equal amount (400 μL) of neutralisation buffer.
- Reseal and rapidly invert 10 times and then spin down briefly using a centrifuge.
- Spin down for 30 seconds at 14,000 RPM (or 20,000 *g*).
- Collect the middle layer of the solution (the bottom layer contains the egg and solid contaminants, while the top layer contains lipid contaminants).
- The DNA (middle layer) can now be used instantly, stored at 4°C for up to a week or stored at -18°C for use later on.

### DNA quality assessments

In order to evaluate the effectiveness of the DNA extraction process and usability of the extracted DNA, a combination of PCR followed by gel electrophoresis and qPCR can be used. A fragment of the Malic enzyme 2 gene (exon 3; 472 bp) was amplified using primers previously designed and validated [14]. This gene was selected due to its well-established availability and its size being within the range of the microsatellites of interest.

- Mix 0.5 μL of sample DNA (middle layer), 3 μL MyTaq HS mix (Bioline, USA), 0.6 pM of each primer (0.12 μL) and 2.26 ultrapure water in PCR tube or plate (10 μL total).
- Perform PCR at the appropriate thermal cycle for gene of interest (in this case, 38 cycles of 95°C for 15 seconds, 60°C for 15 seconds and 72°C for 40 seconds).
- Load 2.5 μL of the PCR product into a 1.25% agarose gel with 5 μL of 1.5× loading dye (ThermoFisher Scientific, UK) in 0.5× TAE buffer.
- Migrate the gel with ethidium bromide and visualised under UV in a transilluminator for the quality of bands and the presence of smear or primer dimer.

The qPCR reactions were run on a QTower 3 (Analytik Jena, Germany) in accordance with the manufacturer’s instructions:

- Mix 1 μL of sample DNA (middle layer), 5 μL Sensifast SYBR No-ROX kit (Bioline, USA), 1 pM of each primer (0.2 μL), 3.6 μL ultrapure water in qPCR plate.
- Perform qPCR starting by 95°C for 3 minutes followed by the appropriate thermal cycle for gene of interest (in this case, 40 cycles of 95°C for 15 seconds, 60°C for 15 seconds and 72°C for 30 seconds).

### Microsatellites validation assessment

A qualitative assessment of the strength of the band was used to determine the amount of PCR product to be added to the capillary electrophoresis (between 0.5 μL and 1 μL).

- Mix required quantity of PCR product with 30 μL of sample loading solution (SLS), and 0.35 μL of size standard (WellRED size standard, Eurofins, Germany) and add to well of capillary electrophoresis plate.
- Top each well off with one drop of mineral oil.
- Run capillary electrophoresis machine (Beckman Coulter CEQ 8000, Beckman Coulter, USA) according to the manufacturer’s instructions.

### Triploidy validation using microsatellites

Once it was determined that the DNA could be used to verify triploidy through fragment analysis of microsatellites, the DNA was sent to Queen’s University, Northern Ireland, for high throughput microsatellite analysis. DNA from 288 78-degree-day eggs and 250 26-degree-day eggs was extracted in the above described manner before being shipped in 96-well plates, where they were tested against the suite of microsatellites (Table 1).

**Table 1:** Microsatellite suite. Set of primers used to verify triploidy in Atlantic salmon eggs.

Microsatellite	Chr.	Forward Primer	Reverse Primer	T_m_ (°C)	Reference
sssp1605UOS	23	GTCTCTCTTCATCCACTGAGGT	ACGCAATGGAAGTCAGTGGA	62	This protocol
sssp2215	24	ACTAGCCAGGTGTCCTGCCGGTC	AGGGTCAGTCAGTCACACCATGCAC	62	[15]
ssad157	26	ATCGAAATGGAACTTTTGAATG	GCTTAGGGCTGAGAGAGGAATAC	62	[16]
ssspG7	29	CTTGGTCCCGTTCTTACGACAACC	TGCACGCTGCTTGGTCCTTG	62	[15]
sssp2216	24	GGCCCAAGACAGATAAACAAACACGC	GCCAACAGCAGCATCTACACCCAG	62	[15]
sssp2001UOS	13	ATCGTCGTGACACAGACAGG	GGCCTGAGATCAGAACAGACC	62	This protocol
EST405	4	CTGAGTGGGAATGGACCAGACA	GTTTACTCGGGAGGCCCAGACTTGAT	62	[17]
SsaD144	3	TTGTGAAGGGGCTGACTAAC	TCAATTGTTGGGTGCACATAG	62	[16]

## Expected results

**Fig 1:**
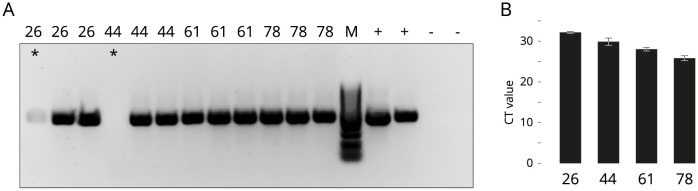
Quality assessments. (**A**). Gel electrophoresis of eggs at 26, 44, 61, and 78 degree-days after 38 PCR cycles. * failed extractions. (**B**) Average CT values for the malic enzyme 2 (n = 10).

To assess the effectiveness of DNA extraction, we employed a combination of PCR followed by gel electrophoresis and qPCR; we amplified a fragment of the Atlantic salmon malic enzyme 2 (exon 3; 472 bp) using primers previously designed and validated [14]. Fig 1A shows the typical outcomes of 38 PCR cycles on eggs at 26, 44, 61, and 78 degree-days.

The cycle threshold (CT values) of preserved eggs at four distinct developmental stages were compared (Fig 1B). In total, 10 eggs from each age group were extracted using the protocol and subjected to qPCR. Levene’s test of equality of variance determined equal variance in CT values, followed by One-Way ANOVA to identify any significant differences between CT values based on egg age. A significant difference was discovered among groups (F(39) = 43.78, p ≤ 0.001). To pinpoint differences between specific groups, multiple comparisons using Bonferroni were conducted, revealing a significant difference in CT values between each group and all others. The efficacy of the extraction was higher in older eggs, with each egg age yielding considerably lower CT values than younger eggs and higher CT values than older eggs.

**Fig 2:**
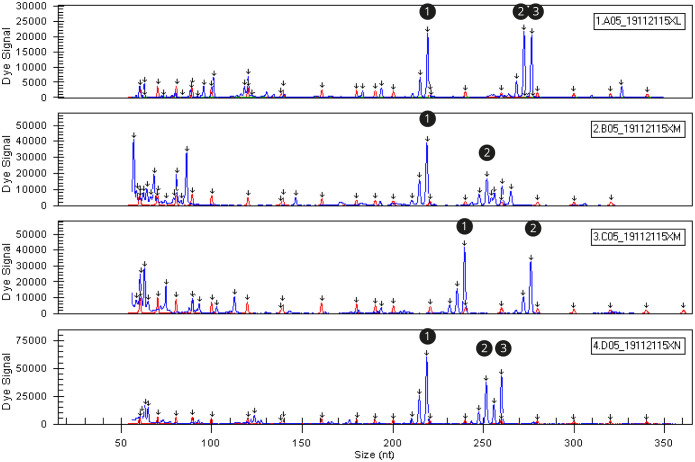
Fragment analysis readout (screenshot). Three distinct peaks (1 and 4) or two peaks (2 and 3) are evidence of triploidy and diploid (and/or homozygous triploid) respectively. Peaks show peaks of fluorescent activity at specific fragment sizes. The early peaks around 50–100 base pairs are PCR errors.

Fragment analysis of the single microsatellite sssp216 demonstrated the potential application of the results for triploidy verification (Fig 2). Samples displaying three distinct peaks indicated trisomy at that locus, providing evidence of triploidy. Not all samples at every locus exhibited three peaks due to microsatellite limitations including stuttering, lack of heterozygosity or lack of chromosomal cross over. Microsatellites with lower heterozygosity or reduced chromosomal crossover would present one or two peaks, even in completely triploid individuals. The rate of heterozygosity of a specific microsatellite will differ depending on the genetic diversity of the population and the parentage of the individual.

**Fig 3:**
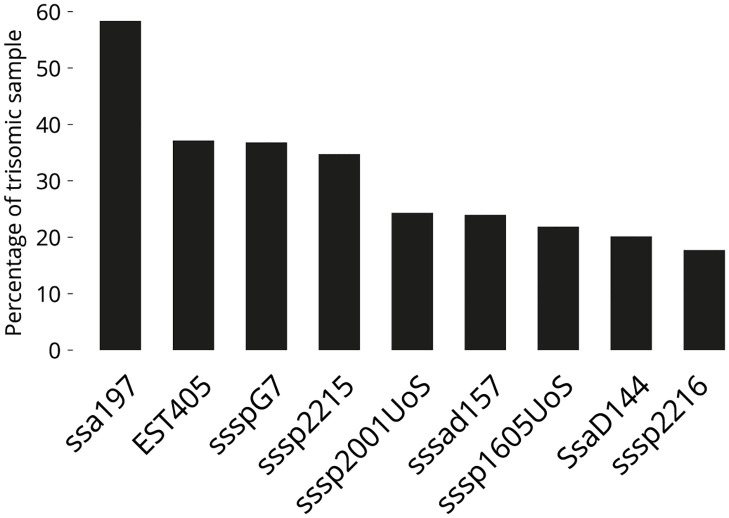
Percentage of individuals showing a trisomic state. At each locus from the suite of microsatellites with the two additional microsatellites (n = 288). 94.08% of individuals showed ≥ 1 trisomic loci, 82.98% showed ≥ 2.

We extracted and validated a total of 288 eggs at 78 degree-days and 250 eggs at 26 degree-days before testing them for the full panel of nine microsatellites in a high-throughput setup at Queen’s University, Belfast, UK. The 26 degree-day eggs did not perform well using this high-throughput method, while the 78 degree-day eggs performed constantly well, indicating that eggs of this age could be validated for triploidy using this DNA extraction technique (Fig 3).

In this study, 94.08% of individuals exhibited trisomy at a minimum of one locus, and 82.98% showed trisomy at two or more loci (Fig 3), by incorporating additional highly polymorphic microsatellites with high crossover, this suite can facilitate the design of suites for use in stocks of alternative genetic origin. The varying utility of specific microsatellites in different stocks is exemplified in the case of microsatellite EST405. A preliminary experiment revealed trisomy in 85.2% of individuals (n = 100) using this microsatellite, whereas the current panel of fish of different genetic origin reported a trisomy rate of 37.15% (n = 288). This difference emphasises the need for an adequate number of microsatellites in identification panels. Larger and specifically validated panels help protect against poor replication or stuttering as well as differences in heterozygosity between different populations or over time, such as where stocks have undergone genetic selection.

## Conclusion

We demonstrated that DNA can be rapidly, inexpensively, and easily extracted soon after fertilisation. This enables triploid verification to be conducted as early as possible, reducing costs and resources associated with triploid production and further enhancing the viability of these sterile fish as alternatives to diploid Atlantic salmon. Additionally, this method offers a DNA extraction technique suitable for various other applications. DNA can be extracted from ethanol-stored samples, and the use of a few basic reagents and laboratory equipment ensures that even the most rudimentary laboratory setups can accommodate this extraction method.

## Supporting information

S1 Protocol Step-by-step protocol, also available on protocols.io. (PDF)

S1 Table Key resources table. (PDF)

S1 Fig Original uncropped unadjusted gel picture (Fig 1A), with annotation. (PDF)
