# Supplementary material for: DNA extraction from recently fertilised Atlantic salmon embryos for use in microsatellite validation of triploidy
Source: PLoS One. 2023 Oct 4;18(10):e0292319. doi: 10.1371/journal.pone.0292319 (PMC10550122; doi:10.1371/journal.pone.0292319)
Supplement: S1 Protocol — (PDF) [file pone.0292319.s001.pdf]

SEP 01, 2023

OPEN ACCESS

DOI:
dx.doi.org/10.17504/protocols.io.kqdg3x93pg25/v1

Protocol Citation: Callum Howard, John B. Taggart, Caroline R. Bradley, Alejandro P. Gutierrez, John F. Taylor, Paulo A. Prodöhl, Herve Migaud, Michaël Bekaert 2023. DNA extraction from recently fertilised Atlantic salmon embryos for use in microsatellite validation of triploidy. **protocols.io** https://dx.doi.org/10.17504/protocols.io.kqdg3x93pg25/v1

License: This is an open access protocol distributed under the terms of the Creative Commons Attribution License, which permits unrestricted use, distribution, and reproduction in any medium, provided the original author and source are credited

DNA extraction from recently fertilised Atlantic salmon embryos for use in microsatellite validation of triploidy

Callum Howard[1], John B. Taggart[1], Caroline R. Bradley[2], Alejandro P. Gutierrez[1], John F. Taylor[1], Paulo A. Prodöhl[2], Herve Migaud[1], Michaël Bekaert[1]

[1]Institute of Aquaculture, University of Stirling, Stirling, United Kingdom; [2]School of Biological Sciences, Queen's University Belfast, Belfast, United Kingdom

Callum Howard: Current Address: AquaBioTech Group, Mosta, Malta;
John F. Taylor: Current Address: AquaMaof Aquaculture Technologies Ltd., Rosh Ha'ayin, Israel
Herve Migaud: Current Address: Mowi Scotland, Glen Nevis Business Park, Fort William, United Kingdom
Michaël Bekaert: michael.bekaert@stir.ac.uk

Michaël Bekaert

ABSTRACT

The current methods used for producing triploid Atlantic salmon are generally reliable but not infallible, and each batch of triploids must be validated to ensure consumer trust and licensing compliance. Microsatellites have recently been shown to offer a cheaper and more convenient alternative to traditional flow cytometry for triploidy validation in a commercial setting. However, incubating eggs to at least the eyed stage for microsatellite validation poses challenges, such as reduced quality and performance of triploids produced from later eggs in the stripping season. To address these issues, we propose another option: extracting DNA from recently fertilised eggs for use in conjunction with microsatellite validation. To achieve this, we have developed an optimized protocol for HotSHOT extraction that can rapidly and cheaply extract DNA from Atlantic salmon embryos, which can then be used for triploidy validation through microsatellites. Our approach offers a simpler and more cost-effective way to validate triploidy, without the need for skilled dissection or expensive kits.

Protocol status: Working
We use this protocol and it's working

Created: Aug 08, 2023

Last Modified: Sep 01, 2023

PROTOCOL integer ID: 86092

Keywords: DNA extraction, DNA quality and quantity assessments, Microsatellites validation assessment, Egg, Tiploid, Salmon

MATERIALS

Consumables

Low throughput:

- 1.5 mL Screw cap tube

High throughput:

- 96-well Clear Round Bottom 2 mL Polypropylene Deep Well Plate
- 96-well Deep well plate seals

Reagents

- NaOH
- EDTA
- Tris-HCl 5 mM pH 8
- Tris-HCl dry
- TAE buffer
- MyTaq HS mix (Bioline, USA)
- Loading dye (ThermoFisher Scientific, UK)
- WellRED size standard (Eurofins, Germany)
- Gel electrophoresis reagents
- 100% ethanol
- ddH_2O

Lab Equipment

- Forceps
- Beakers
- Heat block or laboratory oven
- Centrifuge (capable of 20,000 *g*)
- Gel electrophoresis machine
- PCR machine

Reagent preparation

For 200 mL each alkaline lysis reagent and neutralisation buffer (enough for 500 samples).

Alkaline Lysis Reagent

```
Reagent   Final conc.   Amount for 200 mL
NaOH      25 mM         200 mg
EDTA      0.2 mM        14.88 mg
```

Add ddH2O for final volume of 200 mL. pH will be 12.

Neutralisation Buffer

```
Reagent   Final conc.   Amount for 200 mL
Tris-HCl  40 mM         1.3 g
```

Add ddH2O for final volume of 200 mL. pH will be 5.

DNA extraction

50m 30s

1 If eggs stored in ethanol, remove using forceps and place on clean tissue to remove excess ethanol.

2 Place embryos in a beaker of Tris-HCl ([M] 5 millimolar (mM) , pH 8) for 00:15:00 . 15m

3 Remove the eggs and remove excess liquid with clean tissue.

4 For low throughput needs the eggs can then be placed into individual 1.5 mL screw cap tubes, for high throughput needs the eggs can be places, one per well, into a 2 mL deep 96-well plate.

5 Pierce the chorion by applying pressure using the end of the forceps.

> **Note**
>
> Between eggs, the forceps must be wiped clean before being sterilised using 100% ethanol and ddH_2O.

6 Add 400 µL alkaline lysis buffer to each tube/well and seal.

7 Invert 5 times, and placed into either a heat block or a laboratory oven running at 90 °C for 00:30:00 . 30m

8 Remove and place On ice for 00:05:00 . 5m

9 Unseal and add an equal amount (400 µL) of neutralisation buffer.

10 Reseal and rapidly invert 10 times and then spin down briefly using a centrifuge.

11 Spin down for 14000 rpm, 00:00:30 (or 20,000 g). 30s

12 Collect the middle layer of the solution.

Note

The bottom layer contains the egg and solid contaminants, while the top layer contains lipid contaminants.

13 The DNA (middle layer) can now be used instantly, stored at 4 °C for up to a week, or stored at -18 °C for use later on.

DNA quality and quantity assessments

8m 10s

14

Note

In order to evaluate the effectiveness of the DNA extraction process and usability of the extracted DNA, a combination of PCR followed by gel electrophoresis and qPCR can be used. A fragment of the Malic Enzyme 2 gene (exon 3; 472 bp) was amplified using primers previously designed and validated [1]. This gene was selected due to its well-established availability and its size being within the range of the microsatellites of interest.

Mix 0.5 µL of sample DNA (middle layer), 3 µL MyTaq HS mix (Bioline, USA), [M] 0.6 picomolar (pM) of each primer (0.12 µL) and 2.26 µL ultrapure water in PCR tube or plate (10 µL total).

15 Perform PCR at the appropriate thermal cycle for gene of interest.

15.1 In this case, 38 cycles of
95 °C for 00:00:15 ,
60 °C for 00:00:15 and
72 °C for 00:00:40 . 44m 20s

16 Load 2.5 µL of the PCR product into a 1.25% agarose gel with 5 µL of 1.5× loading dye (ThermoFisher Scientific, UK) in 0.5× TAE buffer.

17 Migrate the gel with ethidium bromide and visualised under UV in a transilluminator for the quality of bands and the presence of smear or primer dimer.

18

Note

The qPCR reactions were run on a QTower 3 (Analytik Jena, Germany) in accordance with the manufacturer's instructions:

Mix 1 µL of sample DNA (middle layer), 5 µL Sensifast SYBR No-ROX kit (Bioline, USA), [M] 1 picomolar (pM) of each primer (0.2 µL), 3.6 µL ultrapure water in qPCR plate.

19 Perform qPCR starting by 95 °C for 00:03:00 followed by the appropriate thermal cycle for gene of interest. 3m

19.1 In this case, 40 cycles of 95 °C for 00:00:15, 60 °C for 00:00:15 and 72 °C for 00:00:30. 40m

Microsatellites validation assessment

20

Note

A qualitative assessment of the strength of the band was used to determine the amount of PCR product to be added to the capillary electrophoresis (between 0.5 µL and 1 µL).

Mix required quantity of PCR product with 30 µL of sample loading solution (SLS), and 0.35 µL of size standard (WellRED size standard, Eurofins, Germany) and add to well of capillary electrophoresis plate.

21 Top each well off with one drop of mineral oil.

22 Run capillary electrophoresis machine (Beckman Coulter CEQ 8000, Beckman Coulter, USA) according to the manufacturer's instructions.
