# Supplementary material for: DNA extraction from recently fertilised Atlantic salmon embryos for use in microsatellite validation of triploidy
Source: PLoS One. 2023 Oct 4;18(10):e0292319. doi: 10.1371/journal.pone.0292319 (PMC10550122; doi:10.1371/journal.pone.0292319)
Supplement: S1 Table — (PDF) [file pone.0292319.s002.pdf]

DNA extraction from recently fertilised Atlantic salmon embryos for use in microsatellite validation of triploidy

S1 Table. Key resources

Consumables

- Low throughput
- 1.5 mL Screw cap tube
- High throughput
- 96-well Clear Round Bottom 2 mL Polypropylene Deep Well Plate
- 96-well Deep well plate seals

Reagents

- NaOH
- EDTA
- Tris-HCl 5 mM pH 8
- Tris-HCl dry
- Gel electrophoresis reagents
- PCR reagents
- 100% ethanol
- ddH_2O

Lab Equipment

- Forceps
- Beakers
- Heat block or laboratory oven
- Centrifuge (capable of 20,000 *g*)
- Gel electrophoresis system
- PCR machine

Reagent preparation

For 200 mL each alkaline lysis reagent and neutralisation buffer (enough for 500 samples).

S1.1. Alkaline Lysis Reagent

Reagent	Final conc.	Amount for 200 mL
NaOH	25 mM	200 mg
EDTA	0.2 mM	14.88 mg

Add ddH_2O for final volume of 200 mL. pH will be 12.

S1.2. Neutralisation Buffer

Reagent	Final conc.	Amount for 200 mL
Tris-HCl	40 mM	1.3 g

Add ddH_2O for final volume of 200 mL. pH will be 5.
