# Supplementary figures and images for: DNA extraction from recently fertilised Atlantic salmon embryos for use in microsatellite validation of triploidy
Source: PLoS One. 2023 Oct 4;18(10):e0292319. doi: 10.1371/journal.pone.0292319 (PMC10550122; doi:10.1371/journal.pone.0292319)

Lanes

X 26 26 26 44 44 44 61 61 61 78 78 78 M + + - - X

Original image

Cropped section  
(Fig 1A)

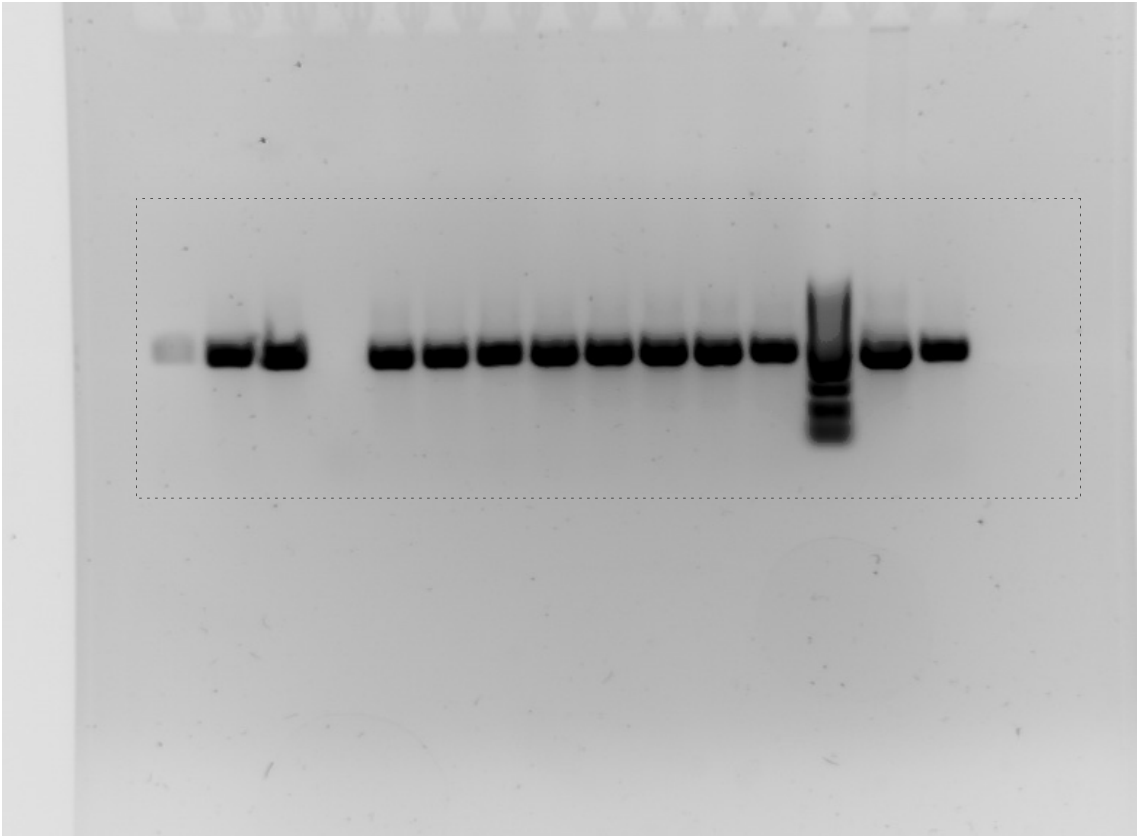

Supplement: S1 Fig — (PDF) [file pone.0292319.s003.pdf]
